# A systematic review of the role of teachers’ support in promoting socially shared regulatory strategies for learning

**DOI:** 10.3389/fpsyg.2023.1208012

**Published:** 2023-09-14

**Authors:** Francesco Sulla, Domenico Monacis, Pierpaolo Limone

**Affiliations:** ^1^Learning Science Hub, Department of Humanities, University of Foggia, Foggia, Apulia, Italy; ^2^Department of Humanities, Pegaso University, Naples, Campania, Italy

**Keywords:** socially shared regulation of learning, co-regulation of learning, social regulation of learning, teacher-students interaction, teacher-students relationship

## Abstract

Interpersonal physiological synchrony and collaboration in educational contexts have been identified as key aspects of the learning environment to foster critical thinking, decision-making, problem-solving, and shared knowledge construction and learning of students. In addition to this, teachers’ support and interaction with students result in a protective factor for students’ well-being and academic outcomes. The main aim of this systematic review was to explore if and how teachers’ support and relationship with students can affect their use of Socially Shared Regulatory Strategies for Learning (SSRL). Studies were identified in six electronic databases (Web of Science, Scopus, PubMed, Embase, and Cochrane CENTRAL and ERIC) following PRISMA guidelines. The initial search yielded a total of 110 records. Fifty-nine studies were fully reviewed, and 16 studies met all inclusion criteria and formed the basis for the review. Studies were analyzed and teachers’ support strategies to enhance SSRL were identified and recorded. This review identifies a range of teachers’ strategies that may foster students’ SSRL, such as prompting and moving from one group to another, helping and checking the groups’ progress, especially in primary and secondary school; flipped classrooms at university level. The results of this systematic review may inform teachers, educational practitioners, the general public and the design of individualized educational interventions aimed at improving teacher-child relationships, their well-being and academic performance.

## 1. Introduction

Michalsky and Cohen (2021) describe the Socially Shared Regulation of Learning (SSRL) as students having similar perceptions and ideologies regarding learning collaborative processes and co-construction of knowledge. In addition, regulation of learning involves individuals controlling their cognition, behavior, motivations, and emotions in learning methods (Zimmerman and Schunk, 2011). SSRL becomes highly necessary due to problems arising during collaborations. According to Hadwin et al. (2018), the interdependence that is observed in SSRL involves knowledge, beliefs, and shared regulatory processes. Processes involved include metacognitive decision-making, evaluation, motivation, strategies, and monitoring. As detailed by Sinha et al. (2015), the relationship between active participation from learners and the regulation process manifesting during the interaction is very close.

Liu et al. (2021) observe how interpersonal physiological synchrony is essential for collaborative tasks, and studies have emphasized its importance for collaborative learning. In this regard, collaboration has been identified as a key aspect of the learning environment to foster critical thinking, decision making, problem-solving, and shared knowledge construction and learning (Liu et al., 2021). This push for increased collaboration is based on the evidence that it is a constructive, social, and active aspect of learning. Success in such groups is usually viewed as wholesome since the students are actively engaged in learning, joint attention, and negotiations from multiple perspectives; thus, their working memory is vastly increased. Grau et al. (2018) posit that collaborations foster self-regulation and the development of an individual’s metacognition in group work. Additionally, Grau et al. (2018) believe that collaboration is an essential skill that needs to be developed during the school years. Interconnections happening in the world today have become a push toward this. Overall, collaboration in academics has been defined as a teaching and learning approach where students have common goals. The targeted goals are worked on together and may include completing tasks, solving problems, or creating a product.

The concept of SSRL is derived from social-cognition literature that entails self-regulated learning (Grau et al., 2018). It includes joint regulations concerning the presented tasks, including learning processes and strategies. In this regard, the control of emotion, behavior, cognition, motivation, and metacognitive monitoring are key processes in social learning regulation. As detailed by Bransen et al. (2022), SSRL was used to explain the regulation of collective learning by groups and their performance. Furthermore, SSRL advocates for the interdependence of group members, and the regulations put up are shared among individuals. This approach focuses on regulation processes within collective learning in the pursuit of harmonized group goals. SSRL is guided by the reciprocated engagements from the group members during regulated operations and activities. Bransen et al. (2022) determined that SSRL is evenly distributed in how its regulations are founded and in the levels of interactions among the group members.

Quackenbush and Bol (2020) note that despite the immense importance of self-regulated learning in students’ academic achievements, it faces the challenge of unintegrated teachers. Teachers can facilitate not only students’ self-regulation, but also their mutual peer-support (co-regulation) and group-level regulation (socially shared regulation of learning); as well as teacher’s practices and their effectiveness may limit the successful implementation of these processes. Therefore, understanding the usefulness of socially shared regulated learning becomes essential. Identification of the emergence of the support from teachers for SSRL is derived from Hadwin’s conceptual framework and Zimmerman’s self-regulated learning theory (Zimmerman, 2008; Hadwin et al., 2018). These theories explain the mechanisms of socially shared regulated learning (Quackenbush and Bol, 2020).

### 1.1. Zimmerman’s self-regulated learning theory

Zimmerman (2008) defines self-regulated learning (SRL) as a student’s conviction of their ability to engage in appropriate behaviors, feelings, and actions in their pursuit of valuable goals while self-reflecting and self-monitoring their progress until they achieve the objective (Zimmerman, 2008). Carver and Scheier (2016) noted that the self-regulated learning theory focuses on the continuous identification and evaluation of progress toward a goal. These processes are measured using feedback from regulations and having self-corrective adjustments. Self-regulated learning processes cover the organization of behavior, feelings, and thoughts in achieving goals. These processes are controlled before, during, and after learning. Other people and the environment provide feedback during self-regulated learning. From the feedback that is given, individuals can readjust themselves in the pursuit of their goals. In return, this gives rise to the learning cycle in which the individual feels motivated to achieve their goals through regulation. Self-regulated learning highlights a person’s behavioral, affective, and metacognitive experiences in achieving their learning goals.

In furtherance of his concept elaboration, Zimmerman (2008) proposes that the first phase of self-regulated learning is forethought. In this phase, the individuals prepare for the learning process, during which tasks to be taken are analyzed and their self-motivation is examined. The performance phase is second in line. Strategies are employed to regulate behaviors, feelings, and thoughts. These strategies are also monitored by individual self-control and self-observation. Additionally, metacognitive strategies are also used to monitor progress and maintain motivation. The last phase is the reflection phase, in which the individual reflects on their actions during the learning process, allowing them to evaluate the overall process, performance, skill mastery, knowledge content, and final products. Zimmerman (2008) has identified the reflection phase to influence motivation for future learning goals and their efficiency.

From Self-regulated learning to Co-regulation and Socially-shared regulation of learning.

Nowadays, the classroom setting has become more active and collaborative. The term “collaborative learning” has been mentioned frequently in the fields of education, and its positive (and negative) aspects have been studied more widely than ever before [e.g., (Van Leeuwen and Janssen, 2019)]. Collaborative learning is a situation in which multiple learners participate in a joint learning activity to expand their existing knowledge together and achieve a common learning goal (Janssen et al., 2012). Learners build a common understanding of the topic and create new knowledge by exchanging their own ideas and negotiating within the group. By sharing the process of reasoning and negotiation, learners become aware of the gap between their thinking and understanding with others, thereby promoting the acquisition of new knowledge and deeper understanding, leading to cognitive development of learners (Teasley, 1997).

Not only cognitive aspects, but also social aspects, are emphasized in collaborative learning. Students are often required to work in a group to study a certain topic and create a learning artifact, such as a final presentation or essay together. Besides creating new knowledge together, students should learn how to collaborate with peers to make a consensus and carry out the study project collectively through the learning processes. As such, collaborative learning requires students to be active in their own learning to contribute to the group learning process, which is in contrast to conventional teaching in which a large amount of knowledge is usually conveyed by the teacher to the students. Each student is responsible for participating in learning activities, adjusting their cognitive, motivational, and emotional states to perform tasks cooperatively with group members.

Although SRL is considered as an individual, invisible, psychological process, one’s regulation can be shared with others by influencing a peer’s regulation processes (co-regulation) or a group’s collective regulation [socially shared regulation; see (Hadwin and Oshige, 2011)]. In the literature, co-regulation is referred to as ‘a transitional process in a learner’s acquisition of self-regulated learning, within which learners and others share a common problem-solving plane, and SRL is gradually appropriated by the individual learner through interactions’ [(Hadwin and Oshige, 2011), p. 247]. Usually, it involves a more capable person such as a tutor, peer or family member who assists in one’s regulation, for example, by explaining the task and checking the deadline of the assignment. Socially shared regulation (SSRL) is defined as ‘the processes by which multiple others regulate their collective activity’ [(Hadwin and Oshige, 2011), p. 253]. In this mode, individual students participating in a collaborative task are responsible for engaging their learning and bringing their own regulation into a group learning situation. Therefore, learning goals and standards are co-created by the members as a group’s decision. The students strategically plan their group learning processes and collectively regulate their performance. Hadwin et al. (2018) stated, ‘What distinguishes socially shared regulation from co-regulation is the extent to which joint regulation emerges through a series of transactive exchanges among group members’ (p. 86); this emphasizes that students’ interactions are the key factor to enable collective regulation of learning among collaborating students.

### 1.2. Hadwin’s regulation of learning

In their study, Hadwin et al. (2018) highlight a need to explore social forms for collaboration and shared knowledge construction through SSRL. Collaborative efforts are given prominence within the group compared to an individual’s motivation, metacognition, or behavior. Co-regulated learning describes a relationship where one individual has more knowledge or skill than the other. During co-regulation, both individuals have to share the regulation of behavior, emotion, motivation, and cognition while pursuing an academic goal. They also have to regulate themselves. Tension observed between the individuals helps shift the ownership of regulation to the group, thereby becoming the SSRL. Ultimately, socially shared regulated learning is the collaborative group work that is achieved through metacognitive control of the task by the group as one. Chan (2012) indicates that the collaborations are made through negotiations, cognitive fine-tuning, and individual’s behavioral, emotional, and motivational states. All of these aspects are necessary to achieve the academic goals that have been set by the group. Interdependence in the performance of individuals within the group is observed while regulation shifts from the individuals to the group. SSRL enables self-regulated learning to have dimensions of learning regulations influenced by others. Such characteristics have become essential in physical and online learning classes because learning is done through cooperation and partnerships.

## 2. Aim

The main aim of this study was to explore if and how teachers’ support and relationship with students can affect their use of SSRL strategies. In particular, specific features of co-regulation related to the teacher-student relationship and whether different strategies are used by teachers in different school grades.

## 3. Methods

### 3.1. Study design

The preferred Reporting Items for Systematic Reviews and Meta-Analyses (PRISMA) guidelines and standards were used to conduct this systematic review. The PRISMA extension used in the preparation of this systematic review was published in the Cochrane Handbook for systematic reviews and interventions (Page et al., 2021a,b).

### 3.2. Search strategy

The databases used to acquire relevant articles for this systematic review included Web of Science, Scopus, PubMed, Embase, and Cochrane CENTRAL and ERIC. Keywords, keyword combinations, field tags, truncations, and Boolean operators “AND” and “OR” were used to search for relevant materials in the databases. Search strings that were used for this study included (“socially-shared regulation” OR “socially shared regulation of learning” OR “socially shared metacognition” OR “socially-shared metacognition” OR “co-regulation” OR “co-regulation of learning” OR “social regulation” OR “social regulation of learning”) AND (“teacher-student relationship” OR “teacher and student relationship” OR “teacher-student interaction” OR “teacher and student interaction” OR “teacher support” OR “teacher practice” OR “pedagogy”). The same search strategy (namely the same string) was replicated across all the databases selected in order to minimize selection bias (Kugley et al., 2016). The search strategy was only adapted depending on the functions within each database in order to search within title, abstract, and keywords: e.g., in Web of science the search string was leaded by ‘TS=’; in PubMed the Advance search functions were used for the same scope. The latest search within the databases was done on the 30th September 2022. The studies identified were then passed through a rigorous process to filter them and acquire substantial and most relevant data for the systematic review. Reviews conducted previously were looked at keenly using a hand search scouring references for valuable studies.

### 3.3. Eligibility criteria

The eligibility criteria used in selecting the articles for the systematic review were agreed upon through consensus from the researchers. The studies eligible for review were empirical studies that included samples composed of teachers and students from every grade and school (from kindergarten to university). Regarding the studied variables, each study should include and measure both SSRL strategies and teachers’ support or relationships with students. The studies to be accepted had no restriction on the design used. Only articles published in English or fully translated to English were considered for inclusion. Moreover, considering the exploratory nature of this review, no restrictions were imposed in terms of the year of publication.

### 3.4. Data selection and data extraction

Rayyan QCRI software was used to manage and screen records (Ouzzani et al., 2016). Duplicates were removed automatically using Rayyan QCRI software, and later two researchers detected and removed possible duplicates manually. After removing duplicates, two authors screened records according to title and abstract’s relevance. Papers considered eligible were analyzed in full text according to the inclusion criteria. A pre-designed standardized excel worksheet was used to extract the relevant data from the studies included in the systematic review. Two independent researchers conducted the extraction of data. Phase one of the extraction process dealt with identifying information, including the year of publication, the author, and the demographic of the population under study. Phase two involved the extraction of the included studies’ results, aims, and conclusions. Then, the two independent researchers analyzed and compared the outcomes of the collected data. Reaching a consensus on the data was key between the two researchers, and where there were disputes, a third party sought to resolve the issues.

## 4. Results

### 4.1. Study selection

**Figure 1:**
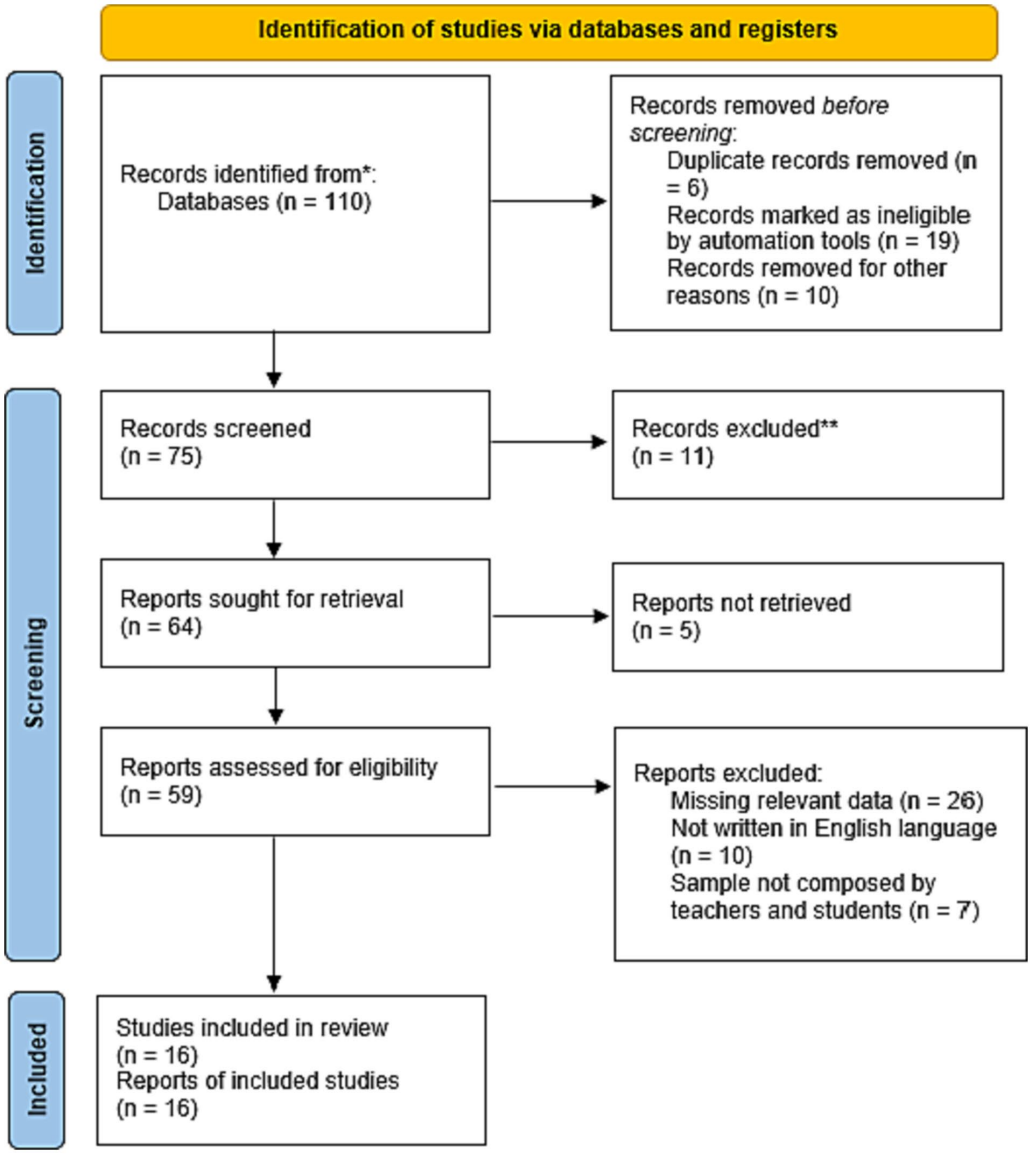
PRISMA flowchart depicting the selection of studies for this systematic review.

From the databases Web of Science, Scopus, PubMed, Embase, and Cochrane CENTRAL and ERIC a total of 110 articles were identified. Search strings were critical in identifying relevant articles in these electronic databases. After the duplicates removal (35 records), 75 papers were assessed according to the relevance of title and abstract. Out of 64 possible eligible articles, 5 were not retrieved and 59 were analyzed in full text for eligibility. Among the eligible full-text papers, 43 records were excluded because they did not meet the inclusion criteria: 26 records were excluded because they lacked relevant data, 10 were not written in English language, and 7 did not involve a sample composed by both teachers and students. Finally, 16 studies remained and were included in the systematic review (see Figure 1).

### 4.2. Study characteristics

Sixteen studies were considered eligible and included in the present systematic review. Table 1 describes the main characteristics (authors, study design, aim and sample) of the included papers, ordered according to sample’s school level. Five studies were conducted on primary school children, from grade 1 to 5 (Kupers et al., 2015; Heritage, 2018; Zhang and Wu, 2020; Hogenkamp et al., 2021; Tao and Zhang, 2021), eight studies on secondary school children (Erdogan, 2018; Järvenoja et al., 2019; Lobczowski et al., 2020; Quackenbush and Bol, 2020; Zakiah and Fajriadi, 2020; Fitriyana et al., 2021; Li et al., 2022; Sobocinski et al., 2022), and three studies involved university students (Yoon et al., 2021; Zheng et al., 2021; Ito and Umemoto, 2022).

**Table 1:** The selected studies’ characteristics, including the author, demographic, aim, and design.

Author	Study Design	Aim	Sample
Kupers et al. (2015)	Mixed-method	Address the co-regulation of student autonomy	38 music students and 8 teachers. Teachers have between 15–46 years of teaching experience
Heritage (2018)	Qualitative	Analyze classroom interactions to obtain evidence of learning to demonstrate that self-regulation is supported through a temporary process of co-regulation between teacher and student in the context of assessment of learning	Students from first and second grade combination class (students aged six to eight) and their teachers
Zhang and Wu (2020).	Mixed-method	This study explores the influence of students’ self-regulatory capacity on the socially shared regulation of learning while collaborating on a data-driven research project	58 undergraduate students
Hogenkamp et al. (2021)	Mixed-method	This study investigates how SSRL manifests itself during cooperative learning using a theory-based approach to identify the prerequisites and consequences of effective SSRL	104 fourth, fifth, and sixth-grade elementary school students
Tao and Zhang (2021)	Mixed-method	Investigate how students and teacher co-organize and sustain didactive activities	43 students of a Grade 5 classroom and their teachers
Quackenbush and Bol (2020)	Mixed-method	Analyze what kinds of teacher behaviors prompt self-regulated learning in students and what kinds of socially-shared regulated learning strategies students use in teams	Participants included 6th to 8th-graders. Classes ranged from 12 to 25 students. Subject areas were mathematics, geometry, and algebra. Two teachers included: Ms. B has 20 years of experience, and Ms. R has 3 years of experience
Li et al. (2022)	Quantitative	This study explores the role of socially shared regulation on computational thinking performance in cooperative learning	94 Chinese middle school students aged between 16 and 18 years
Erdogan (2018)	Quantitative	The aim of this study is to analyze the relationship between students’ self-regulation and their language learning strategies	860 higher education students
Järvenoja et al. (2019)	Quantitative	This study explores how the negative socio-emotional interactions of groups and the related regulation of emotions during a collaborative physics assignment are interconnected with the individual emotional experiences of students	62 higher education students
Zakiah and Fajriadi (2020)	Quantitative	Obtain information on the self-regulated learning of new students in the teaching of mathematics based on the social cognitive perspective	116 high school students
Lobczowski et al. (2020)	Qualitative	Explore how social regulation of learning, scientific argumentation discourse, and socio-emotional interactions occurred and interacted with one another within episodes of collaborative inquiry and discourse	13 high school physics students (grades 10–12) and teachers
Fitriyana et al. (2021)	Mixed-method	Investigate the influences of hybrid learning with videoconferencing and the chemistry android game (chemondro-game) on self-efficacy, self-regulated learning, and chemistry achievement	143 Indonesian eleventh-grade students
Sobocinski et al. (2022)	Mixed-method	How small-scale adaptation (exercising metacognition in the moment) emerges while through monitoring in a collaborative learning settings	12 high school physics students aged 16–17 years
Zheng et al. (2021)	Mixed-mehod	This study aims to provide a personalized intervention for each group participating in collaborative computer-aided learning.	66 college students
Yoon et al. (2021).	Quantitative	Design supports to promote self-regulated learning in flipped classrooms	45 university students
Ito and Umemoto (2022).	Mixed-method	Examines socially shared regulatory processes in peer mentoring. The participants comprise 22 teacher-candidate college students assigned to 11 peer mentoring pairs	22 university students

According to the research methods characteristics, nine studies had a mixed-methods study design assessing the impact of SSRL on students’ autonomy (Kupers et al., 2015), on chemistry hybrid learning and achievement (Fitriyana et al., 2021), and the effects on learning and behavior during collaborative and co-organized activities and tasks (Quackenbush and Bol, 2020; Zhang and Wu, 2020; Hogenkamp et al., 2021; Tao and Zhang, 2021; Zheng et al., 2021; Ito and Umemoto, 2022; Sobocinski et al., 2022). Five studies adopted a quantitative design method exploring the effects of shared regulation on computational thinking (Li et al., 2022), the relation between students’ self-regulation and the individual language learning strategies (Erdogan, 2018), how the negative socio-emotional interactions of the groups could be related to the individual emotions during a collaborative task (Järvenoja et al., 2019), the effects of the self-regulated learning in mathematics (Zakiah and Fajriadi, 2020) and in flipped classrooms (Yoon et al., 2021). Out of sixteen studies, only two were purely qualitative, analyzing classroom interaction according to the temporary process of co-regulation between students and teachers (Heritage, 2018; Lobczowski et al., 2020).

Table 2 summarizes studies’ main findings and outcomes. Results analysis led to identify specific features of co-regulation related to the teacher-student relationship: goal orientation, in terms of focusing on the learning to be achieved; scaffolding, in terms of the assistance that teacher provides to accomplish a goal that is currently beyond students’ efforts; intersubjectivity, a shared understanding based on a common focus of attention; the active construction of knowledge by students, rather than the transfer of knowledge from the teacher to the student; and temporary support, provided through scaffolding and other external supports that students can ultimately appropriate as their own [i.e., (Heritage, 2018)]. Strategies and how teachers and students interact to reach students’ autonomy vary in relation to several factors such as students’ general needs and attitudes toward autonomy and teachers’ way of dealing with students’ autonomous expressions. Highly autonomous students showed different patterns emerging within a lesson compared to low autonomous students. Moreover, students considered ‘talented’ by their teachers seem to be stimulated more by them to increase their autonomous expression, compared to students whose progress was below average [i.e., (Kupers et al., 2015)].

**Table 2:** The Selected Studies’ Characteristics, Including the Author, Intervention, Outcomes, and Results.

Author	Measures	Outcomes	Results
Kupers et al. (2015)	Videorecording and interactions coding, interviews, and questionnaires	Teacher autonomy support, student autonomy expression, teacher motivation, autonomy, progress	Differences in the way autonomy is co-regulated could be in part connected to the general need for autonomy of students; teachers have different ways of dealing with students’ expression of autonomy in lessons; positive relation between amount of out-of-synch per lesson and student motivation and progress
Heritage (2018)	Videorecording and qualitative content analysis using Heritage theoretical framework (2018)	Teacher-students’ interactions in classroom	Specific features of co-regulation were identified: goal orientation, scaffolding, intersubjectivity, the active construction of knowledge by students, and temporary support, provided through scaffolding and other external supports that students can ultimately appropriate as their own
Zhang and Wu (2020)	Observations, videorecording of interactions and coding	Student self-regulation ability, socially shared regulation of learning (SSRL) and data literacy	The results indicated that the group with high SSRL more easily developed a socially shared regulation of learning than the group with low SSRL; high SSRL group performed better in analyzing and creating explanations from the data, as well as evaluating the validity of data-driven explanations, and formulating new questions, two critical components of literacy
Hogenkamp et al. (2021)	Observation and videorecording of code descriptor	Student’s metacognition, cognition, behavior and motivation and their subcategories	Students collectively adopted strategies to regulate group’s metacognition, cognition, behavior, and motivation. No significant differences were found with and without the support regarding the frequency of occurrence of the SSRL subprocesses
Tao and Zhang (2021)	Observation and videorecording analyzed with content and social network analysis	Temporal and interactional processes by which students and teachers co-configured their knowledge	Co-constructed activities with the teacher guide students’ joint attention, participation, and reflection. Social network analysis also showed expansive and opportunistic connections among the students. Moreover, students showed interactive and agentic moves to monitor emerging interests and needs in their inquiry and participate in reflective conversations with their peers and the teacher to expand and reframe their knowledge
Quackenbush and Bol (2020)	Observation and Spruce and Bol’s (2015) observation instrument	Teachers’ and students’ interactions and activities	Teacher vs. student (mean, standard deviation) Forethought and planning: 2.24, 0.52 vs. 0.94 vs. 0.56 Setting goals: 2.37, 0.92 vs. 0.39, 0.65 Seeking information and strategies: 2.09, 0.30 vs. 1.15, 1.52 Allocating resources and team: 2.18, 0.40 vs. 2.15, 1.21 Team and self-instruction: 2.55, 0.69 vs. 0.39, 0.96 Attention focusing: 2.09, 1.22 vs. 0.46, 1.13 Understanding Task Content: 3.82, 0.40 vs. 3.85, 0.38 Reflection and evaluation: 2.02, 0.45 vs. 1.45, 0.85 Strategy use: 3.45, 0.69 vs. 0.85, 1.46 Causal attribution: 0.27, 0.47 vs. 1.85, 1.82 Satisfaction based on performance: 0.91, 1.14 vs. 2.59, 1.44
Li et al. (2020)	Questionnaires and SSRL behaviors were collected through Shimo Docs.	Collaborative self-regulated learning of the students in the classroom	Students of the experimental group significantly outperformed their counterparts both mid-test and post-test. Through this process, students understood what to learn, what tasks to complete, and how to assess their learning progress.
Erdogan, (2018)	Questionnaires	Self-regulations and language learning strategies	The results indicated medium-positive correlations between the two main constructs and further provided evidence for changes in both students’ self-regulation and their language learning strategies based on their achievements and school level
Järvenoja et al. (2019)	Videorecording of lessons and interactions coding	challenges emerging during collaborative learning, co- and socially shared emotion regulation activated in the face of challenges, emotion regulation strategies	General emotional experiences and emotional experiences related to the task are different experiences and contribute differently to socio-emotional interactions during group learning; socio-emotional interactions during learning can influence individual emotional experiences in multiple ways; negative socio-emotional interactions during collaboration did not affect students ‘overall emotional experiences but negatively affected students’ emotions related to the task.
Zakiah and Fajriadi (2020)	Questionnaires	Organizations, elaboration, self-evaluation, learning strategies for examinations, and metacognition	Most students are motivated to learn from their parents, especially from their mothers. The most frequent learning activities of students consist of rereading the subjects already taught. Most students already have adequate facilities for learning at home. Teachers also play a central role in student learning

Results also show that teachers could influence self-regulated learning among students in different ways. Teachers were observed moving from one group to another, helping and checking the groups’ progress. Self-regulated learning in this study reinforced SSRL among the groups, promoted by different behaviors under study [i.e., (Quackenbush and Bol, 2020)]. For example, performance and monitoring behaviors were the most frequent (Quackenbush and Bol, 2020) with a mean (M) of 3.34. Forethought and planning were less frequent with M = 2.24. Based on the teachers’ observation, reflection and evaluation were the least prevalent, having a mean value of M = 2.02. The section encouraged students to scrutinize the effectiveness of their strategies. Behavior was considered when it occurred in the instructional cycle. Students were observed to participate much more in the SSRL. Furthermore, socially Shared Regulated Learning teachers observed learning during engagements with students in class lessons. Planning among students during the forethought phase was difficult. Similar to the teachers’ observation, performance and monitoring were the most frequent among students (M = 2.94), while reflection and evaluation were the least frequent (M = 1.45).

Moreover, during peer group work sessions in the classroom, when the educational content on which students work becomes more challenging, teachers attempt to help students monitor their understanding of the difficult content or create plans to help them complete the task (Lobczowski et al., 2020). These moments are critical, as highlighted by Sobocinski et al. (2022) study because possibilities for adaptation increase as learners become more familiar with the learning content. Finally, Tao and Zhang’s (2021) results showed that co-constructed activities with the teacher guide students’ joint attention, participation, and reflection. In this study, the teacher acted as an attentive listener and observer working to understand students’ diverse ideas, questions, and new progress across individual and collaborative settings, facilitating reflective conversations about evolving goals and inquiry strategies, including ways to address student needs for resources and support.

## 5. Discussion

The main aim of this study was to explore if and how teachers’ support and relationship with students can impact their use of SSRL strategies. In particular, specific features of co-regulation related to the teacher-student relationship and whether different strategies are used by teachers at different school grade.

Results from the systematic review showed that during classroom activities, teachers can use several strategies to interact with their students and foster SSRL strategies for learning. For example, during the self-reflection phase (self-evaluation and self-reaction) teacher prompting and team monitoring are very frequent, and students also engage in shared monitoring behaviors more than in planning behaviors (Quackenbush and Bol, 2020). One explanation for this could be that the planning and evaluation (or reflection) phases required more teacher direction because these strategies were less familiar to students or less likely to occur organically (e.g., goal setting). During the planning and reflection phases, the management of regulation moves back and forth between teacher and student because each participant invites others to recognize their thinking in the context of a specific task. Students are consistently asked to make their judgments about their learning in support of metacognitive capabilities. The teacher’s questions and prompt restatement students to do their thinking. Through the joint responsibility supported by the interaction, the student maintains focus and attention on the task at hand, and the agentive stance of the student is upheld throughout. Co-regulation helps students gradually acquire independent self-regulatory learning processes through which they can develop knowledge, skills, and internal capacities to succeed academically in school and continue future learning for themselves (Heritage, 2018).

The study by Kupers et al. (2015) also shed light on possible teachers’ strategies that foster SSRL but highlights that there is no “one size fits all” approach to students’ autonomy development. For example, what works for a student with a high need for autonomy does not necessarily work for a student with a lower demand for autonomy. Because the need for autonomy is central to children’s healthy development, and because dyadic synchrony has proven to be an important indicator of the quality of interaction between parents and children, it could be possible to speculate that dyadic synchrony between teachers and students in autonomy levels could be positively related with long term student outcomes, as motivation, progress and the overall need for autonomy, which was based on previous literature on this topic (Reeve, 2009; Stroet et al., 2013; Kupers et al., 2015).

Another essential element to consider about SSRL strategies for learning is the difficulty levels of the educational tasks proposed by the teacher to students. The study by Lobczowski et al. (2020) showed the importance of considering difficulty levels as these can affect the student’s ability to regulate, engage in deep discussions and maintain positive socioemotional interactions. They observed that when the educational content of the task became more difficult for students to manage, teachers were more inclined to intervene to support students. These findings reinforce that effective collaborative inquiry requires teachers’ support for how to engage in critical discourse and manage the cognitive and emotional challenges and opportunities afforded by that discourse. Educators are more likely to help their students when they teach them how to engage in inquiry and discourse and how to productively regulate the social interactions and emotions that are endemic to those activities.

Tao and Zhang (2021) with their study showed that age could not be a risk factor for implementing SSRL strategies in class. In this research, also younger students (5th grade) were able to work as epistemic agents to co-construct shared inquiry structures while continually deepening their knowledge in a domain area also thanks to teachers’ support. Teachers and students together engaged in reflectively capturing emergent directions, connections, and patterns of inquiry as they created/adapted shared structures accordingly. Supporting students to enact such transformative agency is essential to dynamic knowledge building that continually unfolds over time, thus breaking traditional classroom barriers and curriculum boundaries. Students co-constructed structures in the form of shared directions and research cycles to organize and guide collaborative inquiry, leading to productive knowledge building. These results support the idea that educators have at their disposal some strategies that may foster students’ use of SSRL strategies, regardless of their age. For example, teachers’ praise exhibited the strongest correlations with students’ SSRL regardless of grade level [e.g., (Guo, 2020; Sulla and Rollo, 2023)]. Moreover, the study by Yoon et al. (2021) included in this systematic review, supported the idea that flipped classrooms – whose effectiveness has been demonstrated in primary and secondary schools [e.g., (Akçayır and Akçayır, 2018)] – are not equally advantageous to all students due to its self-regulated nature, but, was demonstrated to be also effective in fostering SSRL at university level.

Furthermore, as regards tertiary education, differences in educators’ strategies that might foster students’ SSRL might be found in regards to blended learning. In blended/online group learning, or what is called computer supported collaborative learning (CSCL), learners’ interactions are limited because of a lack of social affordances in the digital environment (Kreijins et al., 2002). Learners’ interactions include both teacher–student and peer interactions. Regardless of solo or group learning, the digital environment limits teachers from approaching their pupils to support them and students from seeking help and encouraging each other. Thus, there is a risk of feeling abandoned or isolated without getting any support, which quickly harms students’ learning motivation. However, none of the analysed studies investigated blended/online settings, nor at university level. Future studies should focus specifically on this topic.

That being said, the results of the current systematic review also support the idea that some strategies are more effective than others depending on school levels, but, especially, on students’ needs (again, regardless their age). Indeed, as highlighted by Kupers et al. (2015), when talking about strategies that can foster students’ use of SSRL strategies and, in particular, about dyadic synchrony between teachers and students in autonomy levels, what works for a student with a high need for autonomy does not necessarily work for a student with a lower demand for autonomy. This mean that, in some cases, it might be useful that teachers assess individuals’ abilities and needs of their students in order to tailor their strategies on that particular group. Nevertheless, some strategies are more appropriate for a specific grade level, as, for example, students’ executive functions – and, as a consequence, metacognitive skills that are fundamental to develop good SSRL - are more immature. Indeed, all the analyzes studies that involved primary school students pointed out, for example, the importance of teachers’ prompting [e.g., (Quackenbush and Bol, 2020)].

One study has been found that analyzed the neurophysiological correlates of SSRL strategies used in supportive educational contexts. Specifically, Sobocinski et al. (2022) analyzed heart rate data to measure student’s physiological synchrony. They found that physiological synchrony occurred to the same degree at the beginning, middle, and end of their sample learning session. Group member engagement and sense of togetherness were stable. Physiological synchrony occurs when people interact; in this study, the group members interacted throughout the session.

## 6. Limitations and conclusion

The findings of this review should also be interpreted in light of the limitations of our work. First, we only assessed the English-language literature and may have overlooked significant findings reported in other languages. Second, although we strove to conduct an exhaustive search, a relevant search term may have been omitted, and relevant studies were not retrieved. Third, although we attempted to screen the retrieved studies thoroughly, again it is possible that some salient studies were overlooked. Fourth, even if the analyzed studies covered a good range of school subjects, this study was not able to bring out, in a systematic way, teachers’ strategies that may be effective in fostering students’ SSRL looking at specific disciplines. Future studies should refine the search strategies in order to fill this gap in the literature.

Nonetheless, to the best of our knowledge, this review is the first to systematically review the role of teachers’ support in fostering SSRL strategies for learning including all school levels. Moreover, the study, according to its aims, covered its objectives. Teachers were noted to influence how the Socially Shared Regulated Learning would go. They were observed as catalysts for forethought, planning, performance, monitoring, and evaluation of present tasks. Furthermore, teachers were observed to influence behavior among the participating students through engagement. SSRL is essential for students: it increases motivation, comprehension of subject studies, and positive engagement with the students. Seeing the dearth of research work conducted in these settings, future studies should consider teachers’ supportive role in inclusive special needs education contexts by analyzing SSRL empirically and bringing together different evaluation methods, such as neurophysiological ones, to obtain data on students’ and teachers’ physiological synchrony. This will allow for a better understanding of the mechanisms underlying SSRL strategies and support that can inform teachers, the general public, and possible interventions to foster teachers’ relational and pedagogical skills.

## Data availability statement

The original contributions presented in the study are included in the article/supplementary material, further inquiries can be directed to the corresponding author.

## Author contributions

FS: introduction, methods, discussion and conclusions. DM: methods and results. PL: discussion and conclusions. All authors contributed to the article and approved the submitted version.

## Conflict of interest

The authors declare that the research was conducted in the absence of any commercial or financial relationships that could be construed as a potential conflict of interest.

## Publisher’s note

All claims expressed in this article are solely those of the authors and do not necessarily represent those of their affiliated organizations, or those of the publisher, the editors and the reviewers. Any product that may be evaluated in this article, or claim that may be made by its manufacturer, is not guaranteed or endorsed by the publisher.
